# Enhancing Academic Performance, Cognitive Functions, and Mental Well-Being Through Active Breaks: Evidence from a Pilot Study with University Student Sample

**DOI:** 10.3390/ijerph22111605

**Published:** 2025-10-22

**Authors:** Francesca Latino, Francesco Tafuri, Mariam Maisuradze, Maria Giovanna Tafuri

**Affiliations:** 1 Department of Education and Sport Sciences, Pegaso University, 80143 Naples, Italy; francesca.latino@unipegaso.it; 2 Department of Literature and Cultural Heritage, University of Campania “L. Vanvitelli”, 81100 Caserta, Italy; 3 Department of Medical, Motor and Wellness Sciences, University of Naples “Parthenope”, 80133 Naples, Italy; 4 Department of Literary, Linguistic and Philosophical Studies, Pegaso University, 80143 Naples, Italy

**Keywords:** active breaks, well-being, psychological resilience, executive functions, stress management

## Abstract

Background: Psychophysical well-being, understood as the integrated balance between physical and psychological health, is essential for both personal quality of life and academic performance. Among emerging strategies to support emotional balance and cognitive functioning, active breaks, brief physical activity sessions during study or work, are gaining recognition for their effectiveness. This pilot study explored the impact of active breaks on psychological, cognitive, and physiological variables in a sample of business students, aiming to evaluate their role in enhancing resilience, decision-making, well-being, and autonomic regulation. Methods: An experimental design was used, with students divided into two groups: the experimental group engaged in daily active breaks for 12 weeks, while the control group maintained their regular routines. Psychometric assessments (CD-RISC, DMC Test, PSS, and Stroop Test) and physiological measures (HRV and HRR) were administered before and after the intervention. Results: The findings showed significant improvements in psychological resilience, decision-making ability, and psychophysical well-being in the experimental group. Cognitive performance also improved, as indicated by better Stroop Test scores. Physiologically, increases in heart rate variability (HRV) and heart rate recovery (HRR) suggested enhanced autonomic balance and stress regulation. Conclusions: Active breaks offer a simple and effective strategy to promote students’ holistic well-being—encompassing both psychological and cognitive dimensions—thereby preparing future professionals to manage stress and maintain performance in high-demand environments.

## 1. Introduction

The relationship between physical activity, cognitive functioning, and psychological well-being has attracted increasing attention in educational research, particularly in the context of higher education, where students are required to cope with intense workloads, sustained mental effort, and frequent exposure to stress [1]. In recent years, universities and business schools have begun to recognize that learning cannot be understood solely as a cognitive and technical process but must also be seen as an embodied experience in which physical, psychological, and emotional dimensions play an equally important role [2]. Within this framework, the concept of active breaks, consisting of short periods of structured physical activity integrated into the academic day, has emerged as an innovative approach to enhancing students’ learning capacity, cognitive performance, and resilience. The study of active breaks in educational contexts is part of a broader reflection on how contemporary universities can adapt to the changing demands of society and the labor market, which require not only intellectual competence but also psychological flexibility, emotional balance, and the ability to manage stress effectively [3].

Traditional academic training has long been based on a model that emphasizes theoretical instruction, classroom lectures, and case-based learning, where students primarily engage in passive knowledge acquisition with limited consideration of emotional or physiological aspects of learning. Although effective for transmitting knowledge and developing analytical skills, this traditional approach often neglects the psychophysical and emotional dimensions that sustain learning and well-being over time, thus highlighting the need for more integrative educational models [4]. The growing complexity of organizational life and the increasingly dynamic environments in which university students enrolled in management and business training programs, future professionals expected to assume leadership and decision-making roles, will be called to operate highlight the limits of purely cognitive approaches [5]. Leadership is understood not merely as a formal managerial position but as a multidimensional competence involving self-regulation, emotional intelligence, and adaptive decision-making in complex contexts. Leadership today, shaped by increasingly volatile, uncertain, complex, and ambiguous environments, requires not only the ability to master theoretical frameworks and technical skills but also the capacity to regulate emotions, make informed decisions under pressure, and maintain resilience in the face of uncertainty [6]. Unlike traditional conceptions of leadership focused primarily on authority, control, and cognitive competence, contemporary leadership emphasizes adaptability, emotional intelligence, and the ability to manage stress in dynamic contexts. In this perspective, the integration of physical activity into academic curricula can foster a more holistic development in which body and mind are viewed as interdependent systems that, when nurtured together, enhance learning outcomes.

Recent scientific evidence underscores the profound connection between physical activity and brain functioning [7]. Research in neuroscience has shown that physical activity stimulates neuroplasticity, enhances memory consolidation, improves executive functions such as attention and problem-solving, and fosters the release of neurotransmitters associated with mood regulation and motivation [8]. These findings suggest that the inclusion of structured physical activity within the academic routine can contribute not only to improved physical health but also to superior cognitive performance and more effective stress management. Management students, in particular, often face demanding decision-making and leadership tasks, making them an ideal population for exploring the effects of active breaks on learning and resilience [9].

Recent studies have shown that university students tend to exhibit low levels of physical activity and high sedentary behavior, mainly due to prolonged study hours and screen-based learning environments [10]. According to recent research, more than 60% of university students do not meet the World Health Organization’s recommendations for moderate-to-vigorous physical activity, while daily sedentary time often exceeds eight hours [11]. This behavioral pattern is associated with increased stress, fatigue, and reduced cognitive performance [12]. Therefore, interventions designed to reduce sedentary time and promote regular movement during the academic day are particularly relevant for this population. The implementation of active breaks can represent a practical and effective strategy to mitigate the negative effects of sedentary behavior and foster physical and psychological well-being among university students.

The concept of resilience has become increasingly important in both organizational psychology and educational research. In academic contexts, resilience refers to the ability of students to adapt to challenging circumstances, recover from setbacks, and sustain motivation in the face of adversity [13]. In managerial training, it is closely linked to the capacity of future leaders to make sound decisions under stress, guide teams effectively, and navigate complex organizational dynamics [14]. Physical activity contributes to the development of resilience by strengthening the autonomic nervous system, regulating stress responses, and improving heart rate variability, a physiological marker associated with adaptability and psychological balance [15].

Decision-making quality is another critical dimension that connects physical activity to management education [16]. Decision-making is a complex process that involves both cognitive and emotional components, and under conditions of stress or fatigue, these abilities tend to decline [17]. Physical activity has been shown to enhance executive functioning, support attentional control, and improve emotional regulation, all of which are fundamental for high-quality decisions. In this way, active breaks may offer students effective tools to strengthen their ability to make thoughtful and balanced decisions in both academic and organizational contexts [18,19].

The role of active breaks also extends to the broader domain of mental well-being [20,21]. University students frequently report high levels of stress, anxiety, and burnout, which can undermine academic performance and health. Integrating physical activity into the learning process has been shown to alleviate symptoms of anxiety and depression, improve mood, and foster a sense of well-being that supports engagement with academic tasks [22]. In addition, empirical evidence indicates that brief bouts of physical activity can enhance attention, memory performance, and motivation by promoting neurophysiological activation and reducing mental fatigue [23,24]. In this sense, active breaks represent an important preventive strategy against the mental health challenges increasingly prevalent in higher education [25].

In management education, these findings are particularly significant. Business schools aim to prepare future leaders who must operate in volatile and uncertain environments, such as those shaped by digital transformation, global market fluctuations, organizational change, and the increasing demand for sustainability and innovation, where stress is constant and resilience is essential [26]. The educational paradigm underpinning this study is innovative because it moves beyond the traditional cognitive model of learning and embraces an embodied and holistic approach that integrates physical activity, emotional regulation, and cognitive engagement. This perspective reframes learning as an active, dynamic process that involves the whole person, body and mind, thus redefining how management education can promote adaptability, resilience, and well-being. By integrating physical activity and physiological aspects into management programs, universities can promote a more comprehensive form of education that recognizes the interdependence of cognitive, emotional, and physical well-being [27], thereby enhancing academic performance and preparing students to handle organizational challenges with greater adaptability and effectiveness.

The originality of this research lies in its effort to bring together insights from neuroscience, organizational psychology, and pedagogy to examine the effects of physical activity within the specific context of university education. While much has been written about the benefits of physical activity in general, fewer studies have systematically explored its role within management training programs. By focusing on university students enrolled in management courses, this study addresses a gap in the literature and provides empirical evidence for the integration of active breaks into academic practice.

Finally, the study reflects on a broader transformation in the way education is conceived. For centuries, education has been associated primarily with intellectual and cognitive development, often to the exclusion of the body. This research challenges that dichotomy, proposing instead a vision of learning that integrates physical well-being as an essential dimension. In a world where organizations demand leaders who are not only knowledgeable but also adaptable, emotionally intelligent, and resilient, education must evolve to meet these expectations [28]. The integration of physical activity into academic curricula represents one concrete way in which this evolution can take place, enriching traditional pedagogical approaches with practices that nurture both mind and body.

The present study therefore aims to investigate whether the integration of structured physical activity, specifically active breaks, into management education can improve students’ resilience, decision-making quality, and learning outcomes. More specifically, it seeks to (a) evaluate the effects of active breaks on students’ psychological resilience; (b) analyze the relationship between physical activity and the quality of decision-making under academic stress; and (c) examine whether the inclusion of physical activity contributes to improved learning performance and overall well-being. Based on existing evidence linking physical activity to cognitive and emotional enhancement, it is hypothesized that students who regularly participate in active breaks will demonstrate higher levels of resilience, better decision-making quality, and enhanced learning outcomes compared to those engaged in traditional learning routines. Furthermore, it is expected that these positive effects will be mediated by improved stress regulation and cognitive efficiency.

## 2. Materials and Methods

### 2.1. Study Design

**Figure 1:**
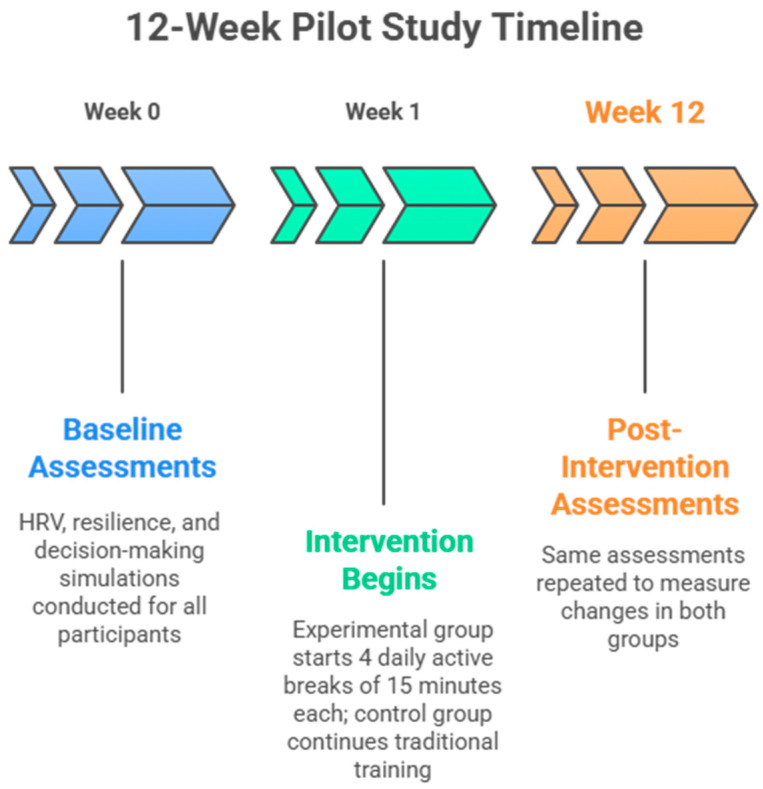
Study protocol.

This pilot study (Figure 1) is a randomized controlled trial with parallel groups and adopts an experimental design to examine the effect of integrating physical activity and physiological aspects into managerial training. A pre- and post-test approach with a control group is used to assess the effects of physical activity on participants’ resilience, decision-making quality, and learning. Participants are divided into two groups: an experimental group that follows a structured exercise program and a control group that does not follow any physical activity during the observation period. Both groups participate in a traditional management training program that includes theoretical lectures, case studies, and business simulations. The research took place over a 12-week span between December 2024 and February 2025, adhering to the ethical principles established in the Declaration of Helsinki and its later amendments. Approval was granted by the Department of Medical, Motor, and Wellness Sciences at the University of Naples “Parthenope” (DiSMMeB Prot. N. 88592/2024). Prior to participation, students provided informed consent.

This study was defined as a pilot study because it aimed to evaluate the feasibility, acceptability, and preliminary effectiveness of an innovative active-break intervention before its application to a larger population. The small and homogeneous sample was selected to test the methodological procedures and identify any potential issues related to implementation or participant compliance. The physical activities proposed were of light to moderate intensity, such as stretching, walking, and breathing exercises, and therefore posed minimal risk to participants. All sessions were conducted under supervision, and students were instructed to perform movements within their comfort level to prevent fatigue or injury. No adverse events were reported during the study.

### 2.2. Participants

The participants in the study are students of 2 management training courses. A total of 60 participants (30 in the experimental group and 30 in the control group) took part in the study. Participants were recruited through an open invitation distributed by the research team, with the support of the academic departments, at the beginning of the semester. All interested students received a detailed description of the study’s objectives and procedures and volunteered to participate.

The sample was selected according to homogeneity criteria such as age, level of managerial experience, and educational background to ensure comparability across groups.

All participants are volunteers and have been informed of the details of the study and the potential risks. These risks were minimal and primarily related to the performance of light to moderate physical activity, such as mild muscle fatigue or temporary discomfort. To minimize these risks, all exercises were designed according to safety standards for healthy adults and supervised by qualified instructors in physical and sport sciences. No psychological or ethical risks were anticipated. Informed consent was granted, and participants were anonymized to preserve privacy. The sample is balanced by gender and geographical origin in order to reduce any cultural bias.

The inclusion criteria for this study required participants to be students enrolled in management training courses from various international business schools. All participants had to be volunteers, fully aware of the potential risks involved in the study, and meet certain homogeneity criteria, including age, level of managerial experience, and educational background. This ensured a more controlled and comparable sample. On the other hand, the exclusion criteria involved excluding anyone who was not enrolled in a management course, those who did not volunteer, or participants who did not meet the homogeneity requirements regarding age, experience, and educational background. These criteria helped maintain the integrity and comparability of the study sample.

A prior power analysis was performed to determine the appropriate sample size for this pilot study, which included 60 participants. Considering the exploratory nature of the research, an effect size of 0.5, an alpha level of 0.05, and a power of 0.80 were used, suggesting a minimum sample of 50 participants. To ensure that the required sample size would still be met in the event of dropouts, an additional 10 participants were included. Randomization was conducted at the class level using a computer-generated random number list, assigning one class to the experimental condition and the other to the control condition. Cluster randomization by class was adopted to avoid contamination effects between participants within the same academic setting, as students from the same class interact frequently and could influence each other’s behaviors if assigned individually. This approach preserved ecological validity and maintained the natural structure of the educational environment while allowing for random allocation at the group level.

### 2.3. Procedure

Students in the experimental group were given four active breaks a day, each lasting 15 min, during which they engaged in targeted physical activities between lessons. Physical activities included aerobic exercises, walking, and light resistance training, while relaxation techniques were implemented as complementary recovery sessions. Exercise sessions are supplemented with mindfulness and breathing practices to improve stress management and promote holistic psychophysical well-being. Participants in the experimental group performed all scheduled active breaks throughout the 12-week intervention. Attendance and compliance were regularly monitored by the research team to confirm that participants in the experimental arm effectively performed the proposed active breaks as planned.

The control group, on the other hand, participates exclusively in the traditional training program without the integration of physical activity. During the entire study period, pre- and post-test assessments are carried out for each participant, including HRV monitoring, psychological assessments (such as the Resilience Questionnaire) and decision-making simulations. Psychological and physiological assessments are administered at the beginning of the study and at the end of the 12-week program.

### 2.4. Intervention Program

The active break-based intervention program (Table 1) is designed to improve physical and psychological resilience, promote overall well-being, and optimize managerial performance through targeted physical activity. Each week, participants are encouraged to spend about 15 min 4 times on active breaks during the working day between classes to counteract a sedentary lifestyle and reduce stress. The breaks are structured to engage both body and mind, stimulating blood circulation, improving flexibility, promoting concentration and reducing psychophysical tension. During the intervention, active breaks were performed collectively at predetermined times coordinated with the course schedule, approximately every 60–90 min of class activity. The 15 min duration of each active break was monitored by the instructor using a professional multisensor sport watch (Polar Vantage V2, Polar Electro Oy, Kempele, Finland). In addition to timekeeping, the device was employed to monitor heart rate in real time, allowing the instructor to ensure that participants remained within the intended light-to-moderate intensity range throughout the sessions. Recorded data were not used for individual analysis but served to standardize exercise intensity and guarantee safety during all activities. Each phase of the routine (warm-up, main exercises, relaxation) was allocated a predefined time segment, allowing for standardized implementation of the protocol. This approach allowed all participants to follow the same routine simultaneously, fostering group engagement, consistency, and adherence to the protocol.

The design of the intervention was based on previous research that demonstrated the effectiveness of brief, structured physical activity breaks in educational and occupational contexts. Several studies have shown that short bouts of exercise performed during study or work hours can significantly reduce sedentary behavior, improve concentration, and promote mental recovery [29,30,31]. In particular, systematic reviews by Infantes-Paniagua et al. [20] and Teuber et al. [22] highlighted that active breaks of approximately 10–15 min, combining aerobic, stretching, and mindfulness components, enhance cognitive functioning, stress regulation, and well-being in university students. The current protocol was therefore adapted from these evidence-based models, with progressive intensity and integration of breathing techniques to optimize both physiological and psychological resilience.

#### 2.4.1. Active Pause Details

Active breaks were integrated throughout the working day, preferably every 60 to 90 min, to prevent sedentary behavior and mental overload. The were performed smoothly and without haste, with participants encouraged to focus on both physical and mental well-being.

#### 2.4.2. Duration and Frequency

1. Each active break lasted 15 min.
2. Breaks were scheduled four times a day at strategic moments during the working day (e.g., mid-morning, after lunch, and in the afternoon).

### 2.5. Measures

#### 2.5.1. Psychological Resilience: CD-RISC (Connor-Davidson Resilience Scale)

The CD-RISC (Connor-Davidson Resilience Scale) [32] is a widely used tool to measure psychological resilience, i.e., an individual’s ability to cope with and overcome difficulties, to adapt to change, and to recover from stressful or traumatic experiences. The test consists of a series of statements regarding inner strength and stress management, to which participants respond on a 5-point Likert scale (from 0 = “Not true for me” to 4 = “Very true for me”). This test will be administered both before the intervention and at the end of the 12-week program to assess the improvement in participants’ psychological resilience. A higher score reflects greater resilience, while changes between pre- and post-intervention scores will provide insight into the effectiveness of physical intervention in improving the ability to cope with difficulties and stress.

The CD-RISC has been validated for the Italian population by Di Fabio and Palazzeschi [33], showing excellent internal consistency (Cronbach’s α = 0.89). In the present study, the scale also demonstrated high reliability (α = 0.87).

#### 2.5.2. Decision-Making Competence: DMC Test

The DMC Test (Decision-Making Competence) [34] measures the ability to make effective decisions, especially in uncertain or complex contexts. This tool focuses on risk management, weighing alternatives, and making informed choices in stressful situations. The test is structured in decision-making tasks that simulate realistic situations, in which participants are called upon to make decisions under conditions of uncertainty. The scores obtained reflect the participants’ decision-making competence, which is assessed on the basis of how rational and appropriate their choices are to the situation. The test will be administered at both the beginning and end of the program to observe any changes in the ability to make informed decisions. An increase in post-intervention scores would indicate an improvement in decision-making competence, suggesting that exercise and psychophysical well-being had a positive impact on decision-making skills. The Decision-Making Competence Test [35] was used following procedures consistent with prior European adaptations for educational contexts. In the current sample, internal consistency was satisfactory (Cronbach’s α = 0.84).

#### 2.5.3. Psychological Well-Being: PSS (Perceived Stress Scale)

The Perceived Stress Scale (PSS) [36] is a tool that measures the level of stress perceived by an individual and how this affects their daily life. PSS explores the perception of stress related to the ability to handle life’s pressures, with questions that relate to the feeling of being overwhelmed or able to cope with difficulties. Participants respond on a 5-point Likert scale (0 = “Never” to 4 = “Very often”). The test will be administered before and after the intervention to measure changes in the level of stress perceived by participants. A higher score indicates greater perceived stress, while a decrease in post-intervention scores would suggest that the physical intervention had a positive effect in reducing psychological stress. This test will help you understand how exercise can help improve psychological well-being by reducing the perception of stress. The Italian validation of the Perceived Stress Scale [37] confirmed good psychometric properties (Cronbach’s α = 0.86). In our study, the PSS also demonstrated adequate reliability (α = 0.82).

#### 2.5.4. Executive Functions and Cognition: Stroop Test

The Stroop Test [38] is one of the most widely used tools for measuring executive functions, such as attention, cognitive flexibility, and the ability to inhibit automatic responses. The test includes three tasks: one for reading words, one for reading colors, and one in which the color of the word and its meaning conflict (e.g., the word “red” written in blue). Participants must respond quickly and accurately, showing their ability to handle conflicting information and maintain attention. This test will be administered both before the intervention and at the end of the 12 weeks to assess whether regular physical activity improves cognitive functions, such as attention and flexibility. Response times and errors made will be measured, with an improvement that would result in greater control of executive functions and better attention management. The Italian version of the Stroop Color–Word Test [39] has shown excellent reliability and validity in assessing executive functions. In this study, internal consistency was α = 0.85.

#### 2.5.5. HRV: HRV Monitoring

Heart Rate Variability (HRV) [40] is an important indicator of an individual’s physiological and psychological health, as it reflects the ability of the autonomic nervous system to respond and adapt to stimuli such as stress. This parameter is closely linked to physical and psychological resilience, as good HRV is an indicator of healthy stress management. HRV was recorded both pre- and post-intervention using a wearable device to measure resting heart rate and post-exercise recovery. The collection of pre- and post-intervention recordings allowed evaluation of whether active breaks improved heart rate variability, indicating better management of physical and psychological stress. An increase in HRV would suggest that exercise had a positive impact on participants’ physiological resilience.

HRV data were collected using a Polar H10 heart rate sensor (Polar Electro Oy, Kempele, Finland) connected via Bluetooth to a compatible recording device. Each participant underwent a 5 min HRV recording in a seated resting position, in a quiet room and at the same time of day (±1 h) for both pre- and post-intervention assessments, following standard short-term HRV measurement protocols. The recordings were analyzed using Kubios HRV Premium software (version 4.0, University of Eastern Finland, Kuopio, Finland). The following time-domain indices (SDNN, RMSSD) and frequency-domain parameters (LF, HF, and LF/HF ratio) were extracted as indicators of autonomic modulation. Artifact correction was performed automatically using the Kubios low-threshold filter, and all recordings were visually inspected to ensure data quality. HRV metrics were computed in accordance with the standards set by the 1996 Task Force (European Society of Cardiology/NASPE) [41], as well as more recent methodological recommendations [41,42,43].

### 2.6. Statistical Analysis

A two-way mixed-design ANOVA (*Group × Time*) was conducted for each dependent variable to test both within-subject effects (pre- vs. post-intervention) and between-group effects (experimental vs. control). This approach allowed for the simultaneous assessment of temporal and group differences.

Effect sizes were calculated using Eta Squared (η^2^) to estimate the proportion of variance explained by each effect, providing an index of the magnitude of the intervention’s impact.

Finally, exploratory Pearson correlations were performed to examine associations between improvements in psychological variables (e.g., resilience, decision-making competence) and physiological indicators (e.g., HRV, HRR) in order to investigate potential psychophysiological relationships underlying the observed effects.

All analyses were conducted using IBM SPSS Statistics version 29.0 (IBM Corp., Armonk, NY, USA). Statistical significance was set at *p* < 0.05.

## 3. Results

### 3.1. ANOVA

To evaluate the effects of the intervention, a two-way mixed-design ANOVA (Group × Time) was conducted on each dependent variable. In addition, Eta Squared (η^2^) was calculated to estimate the proportion of variance explained by each effect, providing a measure of the impact of the intervention. The results are shown below.

Results of the repeated measures ANOVA for each variable (Table 2):

1. Time Effect (Pre/Post): Significant for all variables except the Stroop Test (*p* < 0.05), indicating overall improvements from pre- to post-intervention.
2. Group Effect (Experimental/Control): Significant only for the Stroop Test (*F*(1,58) = 4.26, *p* = 0.041), indicating a difference between groups regardless of time.
3. Group × Time Interaction: Significant for Resilience (*F*(1,58) = 6.16, *p* = 0.015) and approached significance for Decision-Making Competence (*p* = 0.058) and HRV parameters (SDNN, RMSSD, LF/HF ratio) (*p* = 0.058) and HRR (*p* = 0.061), suggesting a trend toward differential improvements in the experimental group.

Eta Squared values for each effect in the variables analyzed (Table 3):

1. Eta Squared indicates the proportion of variance explained by each effect.
2. Effect of Time (η^2^_Time) is the most relevant for Resilience (9%), HRV indices (SDNN, RMSSD, LF/HF ratio) (5.2%), HRR (4.9%), Decision-Making (5.8%) and Well-being (4.3%), confirming improvements over time.
3. Group effect (η^2^_Group) is greater for the Stroop Test (3.5%), indicating differences between experimental and control.
4. Group × Time Interaction (η^2^_Interaction) is Strongest for Resilience (4.6%), with smaller effects for Decision-Making Competence (2.9%) and HRV indices (particularly SDNN and LF/HF ratio) (2.8%) and HRR (2.5%), indicating a trend toward differential improvement in the experimental group.

### 3.2. Pearson Correlation

Pearson correlations were computed to explore associations among post-intervention measures. The correlation matrix (Table 4) shows consistent positive associations across psychological, cognitive, and physiological variables, including HRV indices (SDNN, RMSSD, LF, HF, and LF/HF ratio) and HRR.

**Figure 2:**
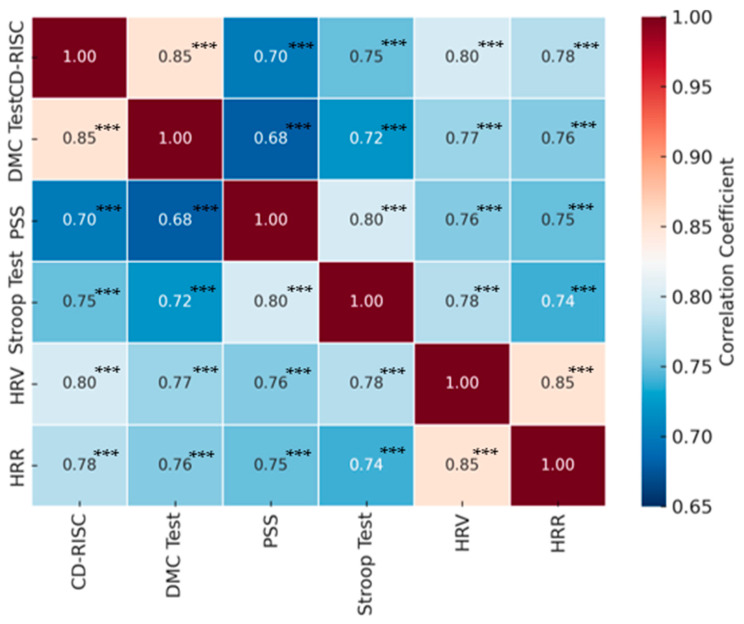
Heatmap of the Pearson correlation matrix. Note: All correlations are positive and significant at *p* < 0.001. Significance levels are indicated as follows: (*p* < 0.05) (*), (*p* < 0.01) (**), (*p* < 0.001) (***).

The heatmap of the correlation matrix indicates that improvements in one area tend to be associated with improvements in the other measures. In particular, increases in SDNN and RMSSD and a decrease in the LF/HF ratio were associated with improvements in resilience and decision-making competence, confirming the link between autonomic regulation and psychological adaptation (Figure 2).

## 4. Discussion

This pilot randomized controlled study sets out to evaluate the impact of active breaks on a set of psychological, cognitive, and physiological variables in a sample of university students enrolled in management training programs. The primary aim was to examine whether the integration of structured, short bouts of physical activity into the educational routine could enhance resilience, decision-making quality, psychological well-being, and physiological regulation. By adopting a pilot design, the study sought to generate preliminary evidence on the feasibility and potential benefits of incorporating physical activity into academic contexts. The results largely supported the initial hypotheses, showing clear improvements in the experimental group across most measures. The effects were statistically significant for resilience and showed non-significant but positive trends for decision-making and physiological regulation, suggesting a potential overall benefit of the intervention. These findings contribute to the growing literature that emphasizes the importance of integrating movement and physical well-being into environments traditionally oriented toward cognitive and professional skill development.

One of the most significant outcomes was the increase in psychological resilience in the experimental group, as assessed by the Connor-Davidson Resilience Scale (CD-RISC). Participants exposed to active breaks reported greater capacity to cope with stress and adapt to challenges compared to the control group, which remained stable throughout the intervention. This result confirms that active breaks provide moments of mental recovery that interrupt stress cycles, thereby strengthening individuals’ ability to manage adversity. The observed improvement is consistent with previous findings that link physical activity to enhanced stress management and resilience [44,45]. In particular, resilience is a fundamental competence in the context of management education, where students are required to develop leadership potential and decision-making abilities in complex and uncertain environments. The ability to recover from difficulties, maintain psychological balance, and face uncertainty with confidence are all qualities that were enhanced in those who engaged in structured physical activity [46]. The absence of similar improvements in the control group highlights the specific role of exercise in fostering these adaptive capacities, beyond what can be achieved through traditional training alone.

The impact of active breaks on decision-making was another noteworthy finding. Decision-making skills, assessed through the DMC Test, showed an improvement trend in the experimental group, which displayed greater reflectiveness and accuracy compared to baseline. Although the Group × Time interaction approached significance (*p* = 0.058), this trend may indicate that active breaks could facilitate more efficient cognitive processing and decision quality, a possibility that should be confirmed in larger samples. The data suggest that active breaks contribute to enhancing the efficiency of cognitive processes, possibly through mechanisms such as increased cerebral blood flow, improved oxygenation, and reduced mental fatigue. Previous studies have emphasized that physical activity improves brain plasticity and neural connectivity, creating conditions that facilitate decision-making [47]. Furthermore, research by Katz et al. [48] and Chang et al. [24] indicated that exercise supports cerebral oxygenation, which enhances attentional resources and decision-making performance. The present study adds to this evidence by confirming that even short and structured breaks during the day can improve the decision-making capacity of students, preparing them to face managerial challenges with greater clarity and composure. In contrast, the control group, which engaged only in traditional academic activities, did not report meaningful changes in this domain, underscoring the added value of active breaks.

The dimension of psychological well-being also deserves attention [49]. Stress, measured by the Perceived Stress Scale (PSS), was reduced in the experimental group after the intervention, while the control group reported no substantial differences between pre- and post-test measures. This result indicates that active breaks offered participants an opportunity for psychological recovery, contributing to better self-perceived well-being [50]. Although our study did not directly measure hormonal or neuroendocrine responses, previous research has suggested that regular and short bouts of physical activity may reduce cortisol levels and promote emotional balance [51,52,53], and may also modulate the hypothalamic–pituitary–adrenal (HPA) axis, thereby decreasing excessive stress reactivity [54]. Our findings are therefore consistent with this literature, even though the precise physiological mechanisms underlying the observed improvements remain to be clarified. Future studies could integrate direct biomarkers of stress regulation, such as cortisol levels or indices of HPA axis functioning, to strengthen the understanding of how active breaks contribute to improved psychophysiological regulation. The control group, deprived of these active recovery opportunities, did not experience comparable benefits, suggesting that the traditional curriculum alone is insufficient to protect students from stress-related difficulties.

Although resilience and perceived stress are conceptually related, their temporal dynamics may differ [55]. The improvement in resilience observed in the experimental group suggests an enhanced capacity to manage adversity and recover from challenges; however, such psychological resources may require time to translate into a subjective perception of reduced stress. In other words, resilience might improve earlier, functioning as a buffer that moderates stress reactivity, whereas changes in perceived stress (as measured by the PSS) could emerge only after a longer exposure to the intervention or in follow-up assessments. This interpretation aligns with longitudinal evidence indicating that resilience often acts as a precursor to stress reduction rather than a direct correlate in short-term interventions [56,57]. Another possible explanation is that the PSS, being a self-report measure of perceived pressure, may be less sensitive to early cognitive and behavioral adaptations elicited by active breaks, which initially affect regulatory and coping mechanisms before impacting overall stress appraisal [58]. Future studies, including delayed post-tests, could clarify whether stress reduction follows the earlier development of resilience.

Executive functions, assessed through the Stroop Test, differed significantly between groups, with students in the experimental condition showing higher inhibitory control and attentional regulation than controls. Students who participated in active breaks showed enhanced inhibitory control and attentional regulation, demonstrating greater capacity to maintain focus and adapt to new situations. These functions are essential in both academic and managerial contexts, as they underlie the ability to regulate behavior, avoid impulsive choices, and engage in reflective reasoning. The improvement can be explained by the increased brain activation associated with physical movement, which stimulates dopaminergic and noradrenergic systems and enhances attentional regulation. Hillman et al. [23] and Best [59] have highlighted that physical activity fosters inhibitory control and cognitive flexibility through the release of neurotransmitters, while Diamond [60] has shown that aerobic exercise strengthens prefrontal cortex connectivity, thereby supporting executive functions. The present study supports these conclusions and demonstrates that even brief and regular sessions embedded in academic life can produce measurable cognitive benefits. Once again, the control group did not show significant changes, underscoring the decisive role of active breaks.

Physiological evidence from heart rate variability (HRV) analysis further strengthened the findings. The experimental group showed an increase in HRV that approached statistical significance (*p* = 0.058), suggesting a possible trend toward improved autonomic regulation, although this finding should be interpreted with caution. This suggests that active breaks may enhance parasympathetic balance and stress adaptability, although further studies are needed to confirm this effect. By contrast, the control group displayed stable levels of HRV throughout the study, suggesting that the traditional academic routine does not promote improvements in physiological regulation. The observed increase in HRV among the experimental group participants is consistent with studies demonstrating that regular physical activity enhances parasympathetic tone and reduces chronic sympathetic activation, thereby improving stress management [61,62,63]. These results provide robust support for the idea that active breaks can promote holistic psychophysical well-being by simultaneously acting on psychological, cognitive, and physiological dimensions.

The combined results of this study have important implications for management education and higher education more broadly. The improvements observed predominantly in the experimental group suggest that traditional training alone may be insufficient to promote resilience, stress management, and optimal cognitive performance. The observed trends, some reaching statistical significance and others approaching it, indicate that even short, structured active breaks can meaningfully contribute to students’ overall well-being [64]. Integrating active breaks into curricula offers a simple yet powerful tool to complement traditional education with interventions that support students’ overall well-being. The development of leaders who are not only knowledgeable but also resilient, adaptive, and capable of making informed decisions requires attention to the integration of physical activity within educational settings. This perspective aligns with recent literature that emphasizes the importance of holistic approaches to leadership training, which incorporate physical, psychological, and cognitive dimensions to prepare individuals for complex organizational environments [65].

Nevertheless, some limitations of the study should be acknowledged. The sample was restricted to a group of university students enrolled in management training, which limits the generalizability of the results to other populations, such as professionals in the workplace or students from other academic fields. The intervention lasted 12 weeks, and although significant improvements were observed, it remains unclear whether these benefits can be sustained over longer periods. Moreover, individual variations in lifestyle and physical activity outside the intervention were not monitored, which may have influenced the results. These limitations suggest caution in the interpretation of findings and highlight the need for further research.

Future studies could expand on this research in several ways. Longitudinal designs would be valuable in determining whether the benefits of active breaks persist over time and continue to influence resilience, decision-making, and well-being in the long term. It would also be useful to replicate the study in other educational and professional contexts, such as healthcare, law, or public administration, to assess the generalizability of the results. Additionally, incorporating technological tools such as wearable devices for real-time monitoring of physiological parameters could provide more precise data on individual responses to active breaks and enable personalized interventions. Research could also explore the role of individual differences, such as baseline levels of physical activity or stress, in moderating the effects of the intervention. Such investigations would contribute to developing tailored strategies that maximize the impact of active breaks on learning and leadership development. Moreover, it would be important for future research to explore the long-term sustainability of the observed effects. Although this study did not include a follow-up phase, examining whether students maintain the behavioral changes introduced by active breaks, such as regular movement, improved stress regulation, and enhanced cognitive functioning, would provide valuable insight into the enduring impact of the intervention. Longitudinal designs could clarify whether these benefits persist beyond the duration of the program and contribute to lasting improvements in lifestyle and well-being.

The findings of the present study may also inform the development of similar interventions for other student populations characterized by high stress levels and demanding academic schedules, such as medical students [66]. Although the structure of the active break program—short, structured sessions combining mobility, strength, and relaxation—can be applied across different contexts, specific adaptations would likely be necessary. For example, in medical education, where students experience prolonged study hours and clinical training, the inclusion of breathing and mindfulness elements may be particularly effective in counteracting fatigue and emotional overload [67,68]. Moreover, scheduling flexibility and shorter session duration could enhance feasibility without compromising effectiveness. In our opinion, the active break model tested in the present study could be successfully replicated in medical education settings, with minor contextual adjustments aimed at addressing the specific cognitive and emotional demands of healthcare training.

Despite these limitations, the strengths of the present study must be emphasized. The use of a randomized controlled design provided methodological rigor, while the integration of psychometric and physiological measures allowed for a comprehensive evaluation of the effects of active breaks. The focus on management students, a group preparing to assume leadership roles in volatile and complex environments, enhances the relevance of the findings for both educational and organizational domains. By demonstrating that structured physical activity can significantly improve resilience, decision-making, executive functioning, and physiological regulation, the study provides compelling evidence for rethinking the design of management curricula.

Taken together, the findings of this study indicate that active breaks represent a promising strategy for enhancing psychological, cognitive, and physiological functioning in university students. While the effects were significant for resilience and showed positive trends for decision-making and heart rate variability, the overall pattern supports the integration of movement-based interventions into academic programs to foster holistic development. The exclusive benefits observed in the experimental group underline the specific contribution of physical activity to mental well-being, cognitive performance, and stress regulation. These results suggest that the integration of active breaks into management education can foster a holistic learning environment that combines body, mind, and knowledge, preparing future leaders to face organizational challenges with greater resilience, adaptability, and decision-making capacity. By demonstrating the pedagogical and organizational value of active breaks, the study opens the way for a reconsideration of educational paradigms, emphasizing the necessity of integrating physical well-being into higher education for sustainable and effective leadership development.

## 5. Conclusions

In recent years, the promotion of psychophysical well-being in educational and professional contexts has emerged as a central concern for both scientific research and organizational practice. The present study indicates that active breaks, when integrated into the academic routine of university students, represent a promising strategy to support psychological, cognitive, and physiological functioning. By focusing on a sample of students engaged in management training, the study highlights how physical activity embedded in learning processes may positively influence decision-making and learning outcomes, thereby enriching the traditional educational approach.

The findings suggest that short periods of structured physical activity are not simply beneficial for physical health but also have measurable impacts on mental performance and adaptive capacities. The pattern of results, significant for resilience and group differences in executive control, with reductions in perceived stress over time and trends for decision-making and autonomic regulation, points to a potential role of active breaks in strengthening skills and resources essential for future leaders. These outcomes align with the broader objective of exploring how physical activity, directly connected to physiological and cognitive functions, can be considered as part of curricula to prepare students for leadership in complex organizational environments. At the same time, the study opens new avenues for future research. It will be important to investigate the effectiveness of active breaks across different academic and professional domains (e.g., healthcare, education, public administration) to verify generalizability. Longitudinal designs will help assess durability of effects, while attention to individual differences (baseline activity, stress) may offer insights to tailor interventions. Moreover, integrating emerging technologies, including wearable devices for real-time physiological monitoring, could enhance personalization of active-break programs.

Overall, the evidence from this trial supports considering the integration of physical activity into management education as a feasible and potentially effective pedagogical innovation, fostering a more holistic educational experience that combines cognitive, emotional, and physiological dimensions. While not all outcomes reached conventional significance, the consistent pattern, significant improvement in resilience, group differences in executive function, reductions in perceived stress, and trends for decision-making and HRV, support the inclusion of movement-based practices to equip future managers with resilience, decision-making quality, and adaptability in volatile and uncertain contexts. These results support the consideration of integrating physical well-being into academic training as a component of sustainable and resilient leadership development.

## Figures and Tables

**Table 1:** Program Structure.

Weeks	Main Objectives	Exercises
Week 1–4: Introduction to Active Breaks and Mobilization	- Introduction to active pause. - Development of body awareness and muscle relaxation. - Improved posture and blood circulation.	Stretching and Neck Mobilization (5 min) - Lateral tilts of the head (left/right). - Slow head rotations (clockwise/counterclockwise). - Shoulder tension and relaxation. - Trapezius stretch. Mobilization of the Spine (5 min) - Seated torso rotations. - Spine extensions (arched/round back). Upper and Lower Extremity Stretching (5 min) - Biceps and triceps stretching. - Calf, quadriceps, and glutes stretching. - Light lunges for hip mobilization.
Week 5–8: Intensification and Integration of Breathing	- Increase exercise duration and intensity. - Integrate breathing techniques to reduce stress and improve concentration. - Increase light muscle strength.	Dynamic Stretching and Light Cardio (10 min) - Jumping Jacks (30 s). - High Knees (30 s). - Butt Kicks (30 s). - Quadriceps and calf stretch. Breathing and Relaxation Techniques (5 min) - Diaphragmatic breathing (4 s inhale/exhale). - Progressive muscle relaxation (feet to head). Muscle Strengthening (5 min) - Bodyweight squats (30 s). - Push-ups (30 s). - Plank on elbows (starting at 10 s, increasing progressively).
Week 9–12: Optimization of Physical and Psychological Resilience	- Work on physical and mental endurance. - Increase exercise difficulty. - Introduce coordination and balance exercises for stress management.	Intense Cardio and Interval Training (10 min) - 30 s circuit with 10 s rest: - Jumping Jacks - Alternating lunges - Mountain Climbers - Jump Squats Muscle Strengthening and Balance (5 min) - Side plank (30 s per side). - Single-leg squat (30 s per side). - Glute Bridge (30 s). Advanced Breathing and Mindfulness (5 min) - Square breathing (4 s inhale, 4 s retention, 4 s exhale, 4 s pause). - Mindful movement (slow walking, focusing on breath and movements).

**Table 2:** Results of repeated measures ANOVA for each variable.

Variable	F (Group)	*p* (Group)	F (Time)	*p* (Time)	F (Interaction)	*p* (Interaction)
CD-RISC	0.21	0.646	12.08	0.0007 *	6.16	0.015 *
DMC Test	0.55	0.461	7.39	0.0076 *	3.67	0.058
PSS	1.60	0.208	5.37	0.022 *	2.11	0.149
Stroop Test	4.26	0.041 *	1.01	0.316	0.42	0.516
HRV	2.71	0.102	6.65	0.011 *	3.65	0.058
HRR	2.54	0.116	5.92	0.018 *	3.42	0.061

Note: *p* values marked with an asterisk (*) indicate statistically significant results (*p* < 0.05). A trend toward significance was observed for Decision-Making Competence and HRV and HRR (*p* ≈ 0.06).

**Table 3:** Eta Squared values for each effect in the variables analyzed.

Variable	η^2^ (Group)	η^2^ (Time)	η^2^ (Interaction)
CD-RISC	0.0016	0.0898	0.0458
DMC Test	0.0043	0.0579	0.0288
PSS	0.0128	0.0429	0.0168
Stroop Test	0.0350	0.0083	0.0035
HRV	0.0210	0.0515	0.0283
HRR	0.0194	0.0490	0.0252

**Table 4:** Pearson correlation coefficients between each pair of post-intervention variables.

Variable	CD-RISC	DMC Test	PSS	Stroop Test	HRV	HRR
CD-RISC	1	0.85	0.70	0.75	0.80	0.78
DMC Test	0.85	1	0.68	0.72	0.77	0.76
PSS	0.70	0.68	1	0.80	0.76	0.75
Stroop Test	0.75	0.72	0.80	1	0.78	0.74
HRV	0.80	0.77	0.76	0.78	1	0.85
HRR	0.78	0.76	0.75	0.74	0.85	1

Note: All correlations are positive and significant at *p* < 0.01 or *p* < 0.001.

## Data Availability

The data presented in this study are available on request from the corresponding author. The data are not publicly available due to privacy restrictions.
